# Storying My Lifestyle Change: How Breast Cancer Survivors Experience and Reflect on Their Participation in a Pilot Healthy Lifestyle Intervention

**DOI:** 10.1080/17482631.2020.1864903

**Published:** 2021-02-21

**Authors:** Shira J. Yufe, Karen D. Fergus, Dana A. Male

**Affiliations:** a Department of Psychology, York University, Toronto, Ontario, Canada; b Odette Cancer Centre, Sunnybrook Health Sciences Centre, Toronto, Ontario, Canada; c Department of Psychosocial Oncology, Tom Baker Cancer Centre, Calgary, Alberta, Canada

**Keywords:** Breast cancer, survivor, healthy lifestyle, group intervention, narrative

## Abstract

**Purpose**: Healthy lifestyle interventions after breast cancer treatment have generally been studied in terms of weight-loss outcomes, which leaves a gap in our understanding of the phenomenological experience of such programs. Our knowledge of *how* or *why* women recovering from breast cancer engage or do not engage in these programs is limited. Thus, we aimed to share subjective experiences of lifestyle change within a 12-week group intervention entitled “Healthy Lifestyle Modification After Breast Cancer” (HLM-ABC).

**Methods**: The present research entailed a multiple case study of four breast cancer survivors who participated in the HLM-ABC. Participants were interviewed longitudinally at four time-points: (1) pre-intervention; (2) mid-way intervention; (3) post-intervention, and (4) three-months post-intervention.

**Results**: We analysed storytelling of participation in the HLM-ABC program to investigate participants’ unique and gradual endeavours towards living a healthier lifestyle. A qualitative, narrative analysis was applied to each participant’s set of interviews, which yielded two distinct story-telling patterns while participating in the HLM-ABC program: one “plot-driven” and one “character-driven”.

**Conclusions**: These two narrative styles appeared to correspond with differing levels of intervention uptake and perceived success in the program. The implications of these narrative styles and their relationship to healthy lifestyle intervention are discussed.

## Introduction

Due to the global surge in breast cancer (BC) survivorship (World Cancer Research Fund International, 2014), interventions for this population are becoming increasingly relevant and studied. Weight loss and exercise programs, in particular, are receiving much attention due to the mounting evidence that being overweight or obese increases the likelihood of BC cancer recurrence and all-cause mortality (Neil-Sztramko et al., 2019; Rock et al., 2015). It has been demonstrated that, compared to non-obese survivors, the risk of cancer recurrence is doubled among BC survivors who are obese (Kamineni et al., 2013). Over 50% of women may gain weight during and after treatment (Vance et al., 2011) and an even greater number of women diagnosed with BC experience pre-morbid obesity (Rock et al., 2013). Scientists speculate that the excess adiposity observed during survivorship may be due to hormonal changes induced by treatment (i.e., hormone therapy) (Carmichael, 2006; Villarini et al., 2012).

Many BC survivors seek out information on the secondary prevention of BC in an effort to stave off recurrence and improve their overall health (Green et al., 2014; Kwok et al., 2015), yet oncology healthcare providers’ recommendations to increase physical activity and monitor food consumption are often not sufficient to inspire these changes (Park et al., 2015). Cancer survivors in general, including those who have had BC, may engage in routine preventative care, but are not as likely to meet health behaviour recommendations (Lemasters et al., 2014). In order to flexibly respond to lack of adherence to survivorship health recommendations, there has been a noticeable transition in the literature from the study of obesity as a risk factor for cancer recurrence towards the development and investigation of interventions that encourage the establishment and long-term maintenance of healthy lifestyle habits and normal body mass index through routine physical activity and consumption of nutritious foods (Hamer & Warner, 2017; World Cancer Research Fund International, 2014). Not only have such efforts been demonstrated to reduce risk of recurrence, but they are also related to the prevention of other chronic conditions (Rock et al., 2013). Healthy lifestyle interventions for BC survivors play an important role in promoting, encouraging, and supporting such behaviours.

The majority of structured programs target either dietary or physical activity changes (Demark-Wahnefried et al., 2012). However, in recent years there has been an increase in more integrative lifestyle interventions that target different mind-body factors with potential impacts on both weight-loss and quality of life such as motivation, self-efficacy, yoga, sleep, and meditation (Arun et al., 2017; Larkey et al., 2014; Lyman et al., 2018; Reeves et al., 2014). One comprehensive review on the effectiveness of such interventions identified several characteristics of more successful lifestyle trials (O’Connell et al., 2016). This large-scale report found that changes in dietary behaviour have a higher likelihood of prolonged maintenance over physical activity. Research trials that targeted individuals with BC while they were undergoing treatment had a more optimal outcome than lifestyle interventions that were timed for after treatment, as measured by behavioural maintenance (O’Connell et al., 2016). Individual characteristics such as age, pre-intervention BMI, and duration of the intervention did not prove to be significantly meaningful variables between more or less successful trials.

Collaboratively developed by oncology clinicians at York University and the Odette Cancer Centre, a pilot intervention entitled the Healthy Lifestyle Modification After Breast Cancer (HLM-ABC) program addresses the need for a more integrative psychosocial lifestyle modification program (Yufe et al., 2019). The HLM-ABC is a professionally facilitated, group-based intervention for BC survivors that has been piloted using both in-person and online formats. Participants were women who gained weight after BC treatment or were obese prior to their diagnosis. The aim of the program was to promote and learn healthful habits tailored to the womens’ individual health goals and readiness for change. This program model takes a biopsychosocial perspective and draws from the transtheoretical model of behaviour change to recognize stages of behavioural change (Prochaska & Velicer, 1997), an intuitive eating approach to teach participants to attend to internal hunger and satiety signals (Tribole & Resch, 2012), and supportive-expressive group therapy principles to afford participants a safe context to explore and express their feelings and thoughts about behaviour modification and how these relate to their existential concerns and sense of personal control (Kissane et al., 2007; Tabrizi et al., 2016). Importantly, psychosocial outcomes are not well-studied or understood among BC survivors participating in structured programs designed to improve their overall health (Reeves et al., 2014). This identified gap in the literature calls for a deeper look into the experiences of BC survivors as they attempt to make health behaviour changes during this period of survivorship, when recurrence is typically top-of-mind.

### Objectives

The paucity of qualitative research among breast cancer survivors enrolled in healthy lifestyle interventions has led to a lack of knowledge of the complex process of health behaviour change among this group, despite such interventions being well-studied quantitatively. In order to understand the subjective experiences of women partaking in lifestyle modification interventions, a qualitative, narrative analysis was used. A narrative approach permitted an in-depth elucidation of how these women reacted to the intervention, and how they attempted to make lifestyle changes. A longitudinal interview design was applied in order to capture a clear and vivid course of attempting to make health behaviour changes over time, throughout the course of the intervention and thereafter. To capture individual trajectories of change, we turned to the women’s narratives, with attention to their engagement with the intervention as well as their story-telling style. The process of “storying” during or after a cancer diagnosis can provide important information into the post-illness experience (Frank, 2013). In essence, we sought to understand how BC survivors narrated their journeys throughout the HLM-ABC program in order to inform our understanding of lifestyle modification processes in this population.

## Methods

### Participants

BC survivors who received treatment at the Odette Cancer Centre at Sunnybrook Health Sciences Centre in Toronto, Canada were recruited by healthcare providers if they were diagnosed with a primary BC (stages I–III), completed their treatment—including surgery, chemotherapy, and radiation within the last 5 years—irrespective of continued Herceptin or hormonal, pharmacological treatment. Prospective participants were asked by their providers whether they consented to be contacted by the research coordinator for a brief screening interview to confirm eligibility. The research coordinator assessed individual weight gain post-treatment and comorbid conditions. Participants were eligible if they self-reported a five pound or more weight increase after treatment, or had an overweight status (BMI ≥ 25 kg/m^2^) since or before their BC diagnosis. Participants were also asked to identify any other comorbidities such as ongoing physical or mental health conditions and were eligible if these health concerns were being managed.

### Data collection

Four women met the eligibility criteria and participated in a pilot HLM-ABC intervention over a period of 4 months in 2015. Participant demographics are detailed in a published study of narrative themes that emerged from this dataset (Yufe et al., 2019). This pilot program was delivered in-person over 12 weeks, in the format of 90-minute sessions, at Wellspring Cancer Support . Each session was co-facilitated by a registered psychologist and a trained volunteer BC survivor, wherein participants engaged in group discussion, received psychoeducation, and reviewed weekly homework assignments (Yufe et al., 2019).

To capture active storying of health behaviour change as it unfolds, participants were interviewed on four different occasions—(1) pre-treatment, (2) mid-way through treatment, (3) post-treatment, and (4) three-months post-treatment. The interviews were conducted in a semi-structured, iterative fashion in that the interview protocol was modified from one time point to the next in order to accommodate recurring narrative storylines and broader themes. Still, interview questions were treated as a guide, rather than mandatory questions, in order to have made room for a narrative-style interview where participants were given sufficient freedom to narrate as they wished (Josselson & Lieblich, 1999). Table I shows examples of interview questions from each interview time point. All interviews were audio-recorded and then transcribed by a research assistant with ExpressScribe software. Interview transcripts were managed with HyperRESEARCH (Version 3.7.1).

**Table I.** Semi-Structured Interview Questions

Interview Time Point	Sample Interview Questions
Pre-Intervention Interview	Do you consider yourself to be someone who leads a healthy lifestyle? Explain. How do you feel about your weight and body? Did this change at all after having undergone breast cancer treatment? Can you recall any event in your past that you believe impacted your relationship to your body, body image or self-esteem? Eating/food? Physical activity? Is there anything you would like to share, that you think might help us better understand you and how to best support you in achieving your lifestyle goals throughout this program?
Midway-Intervention Interview	How are you finding the group thus far? Are there any particular insights that you have gained from participating thus far? (Probe about relationship to self, body, food) How do you feel about your weight and body at this moment in time?
Post-Intervention Interview	At this point, do you consider yourself to be someone who leads a healthy lifestyle? Have you noticed a change in your lifestyle after participating in the group? How would you describe your current lifestyle? What was it like to take part in this program? What were your expectations going into the program? (probe re: whether these were met). In what way did the program fall short of your expectations?
Three-Month Post-Intervention Interview	Did the program help you maintain any changes now after 3 months? Please elaborate. Which session or group topic did you feel has helped you the most after the intervention finished? If you can think back to the pre-treatment interview and your expectations of the intervention, do you think you were successful in achieving in what you were most hoping to achieve(i.e., what were your goals)? Do you consider yourself to be someone who leads a healthy lifestyle? Please explain.

*Examples of interview questions extracted from the semi-structured interview guide.

### Analysis

The interview data were analysed narratively to capture each woman’s idiosyncratic process of change or non-change in relation to the intervention. We endeavoured to look for variation amidst narrative tellings (in terms of style and content), in seeking qualitative “integrity” to the phenomenon (Levitt et al., 2017). Integrity guidelines, or degree of rigour and trustworthiness, ensure that the author is sufficiently close to the data, and the methods used are effective for the research endeavour (Levitt et al., 2017). Central to our research aims, we cycled between analysing parts and the whole of the interviews, as recommended by Levitt et al. (2017). In addition, we aimed to conduct rich interviews that would yield detailed and complex descriptions, with the potential for multiple subplots and layered meanings (Randall et al., 2015) and that could be studied both within each case and across each case (Gustafsson, 2017).

Each woman’s set of four interviews were read sequentially and linearly as a distinct narrative (Hoshmand, 2005) resulting in a multiple-case study articulation (Yin, 2014).

Understanding the intricate relationship between the parts (individual interviews) and the whole (set of four interviews per participant) was integral to our research question of how BC survivors narrate and parcel together their experiences of participating in a healthy lifestyle intervention (Josselson, 2013). The narrative analysis was adapted from Randall et al.’s (2015) narrative framework and Kohler-Riessman’s method (Kohler- Riessman, 2008). These methodological guides were used in tandem to assess the narrative content and structure contributing to the dimensionality apparent in each case. The first author read each interview and whole narrative set (4 interviews per individual) numerous times to identify narrative features. For example, instances of narrative complexity were coded using some of the following integral narrative questions, as adapted from Randall et al.’s narrative guidelines (Randall et al., 2015): Is the story thick or thin with description? What is the autobiographical reasoning for perception of success or failure in one’s change (or no change) trajectory? In this analysis, the relative degree of presence was coded as a narrative feature over and above simply presence or absence of a narrative feature. The researchers then came together to discuss the narrative features vis-à-vis the transcripts and to examine each interview content across time-points for linear progression, narrative arcs, and protagonist agency (See Table II for an overview of narrative features and their definitions). The narrative features used for coding were part of an a priori framework for coding. Finally, the research team discussed and debated these until a consensus was reached.

**Table II.** Narrative construction features*

Narrative Feature	Description
*Narrative Complexity*	Generality vs. specificity (of details) Degree of detail and dialogue
*Narrative Arc*	Beginning-middle-end structure (linear or not) Shape of the narrative Rising and falling actions
*Narrative Tone*	The genre/pervasive feeling that runs through what is said (e.g., tragedy or adventure, hopelessness, despair, optimism, excitement, etc.)
*Protagonist Agency*	The degree of agency or authorship they possess in their self-characterization Sense of control vis-à-vis events in their life/world Internal vs. external locus of control
*Characterization*	The way they characterize themselves (e.g., victim, hero, etc …) Self-portrayals vis-a-vis existing archetypal characters
*Autobiographical reasoning*	The degree of sophistication and connection of events (e.g., part-whole, event-life)
*Narrative openness vs. foreclosure*	Views of the future and past events How is the author operating in reference to the past, present, future? Sense of dwelling on the past or hopefulness/hopelessness about the future
*Master narratives*	References to larger stories (e.g., religion, culture, or family) with which they identify and inform the meaning-making process

*Narrative features adapted from Randall et al. (2015)

## Results

Pseudonyms were assigned to each participant and minor demographic details were altered to ensure the identities of the participants remained anonymous. Two distinct styles of story-telling emerged from the analysis of the participant BC survivors’ narratives that we named: (1) plot-driven and (2) character-driven. Keeping in mind that the context of this narrative analysis is an intervention intended for healthy lifestyle change, the researchers defined a story “outcome” or “ending” as change in behavioural habits, notwithstanding other types of changes that may occur within an intervention, for example, personal growth or self-acceptance to name only a few. The distinction made between plot- and character-driven narratives is based on the following, primary criteria: Degree of linearity or non-linearity, and degree of agency and non-agency expressed. By linearity we mean a clear, beginning-middle-end structure. In the context of the HLM-ABC, the plot-driven narrative possesses a linear plotline with character agency and corresponded with the following features (as determined by Randall’s guidelines and the researchers of the present study): satisfaction with the program, perceived change in oneself or one’s body, change processes that are evident to the researcher, sense of hopefulness, and external conceptualizations of shifts or change (e.g., keeping a workout plan). On the other hand, the character-driven narrative contained a non-linear plotline and lack of character agency and corresponded with features of: narrative foreclosure, a sense of hopelessness, stability of character, lack of perceived change in one’s health behaviour, narrative-interruptions in the participant’s account of their experience, and/or internal conceptualizations of mild or small shifts of change (e.g., cognitive restructuring). At their core, plot-driven narratives were stories of movement and resolve, whereas character-driven narratives were stories of relative stagnancy, with self-identified behaviour change as our metric or marker of transformation.

Two of the BC survivors, Louise and Felicia, seemed to narrate in a character-driven way that was less linear in its progression and focused on their personal characteristics over and above the task of lifestyle change. Melissa and Nicole, on the other hand, narrated in a plot-driven way where a clear beginning-middle-end structure was evident, generally resulting in perceived changes and healthy habit formation. Each style of story-telling is presented below and each woman’s case is categorized by narrative style. First, Louise and Felicia are presented as prototypical character-driven narratives, followed by those of Melissa and Nicole which are presented as prototypical plot-driven narratives.

### Character-driven narratives: Louise and Felicia

#### Louise

Louise’s narrative and self-characterization centred on defeat and hardship as a BC survivor. She maintained a “life is tough” attitude and stated that she has learned that tragedy follows her. Louise’s rare autoimmune disease and her genetic risk for cancer appeared in her narrative as a “continuous plotline.” In other words, Louise felt she had a grim, genetic destiny, which contributed to the “narrative foreclosure” (Freeman, 2001) present throughout her story.

During her pre-treatment interview, she described a sense of hopelessness: “I’m just tired, exhausted, lazy, lacking motivation, and I also found out too that I have low iron, so that’s my excuse. Sometimes … I just get so down and I can’t, and I don’t do anything you know.”

Further storying a sense of narrative foreclosure, Louise’s storytelling possessed a non-linear structure with some (albeit few) “narrative arcs” or changes in her attitude or characterization over time. In other words, her story lacked a clear beginning-middle-end structure. For example, Louise would at times comment on a slight improvement, but then swiftly harken back to what appeared to be a fatalistic perspective. Midway through the intervention, Louise mentioned that she tended to eat when she felt bored and noticed that eating breakfast made her “less starving throughout the day,” yet denied learning new insights about her health behaviours or deriving benefit from the HLM-ABC. She would also move quickly between a more serious tone and a joking, lighter narration. When asked to recall any events or formative relationships that impacted her relationship to her body image, she spoke of a cousin who bullied her, calling her “fat and ugly.” Not only did Louise seem to internalize these harsh comments, earlier referring to herself as both of these things, but there appeared to be an express need to cope with these difficult memories related to her body, with food as a soother, as she recalled such during the interview. She stated directly to the interviewer:

P1: I think I’m going to go eat something after this! [laughing]

I: Oh boy.

P1: I have kale juice, so you’re okay

I: Okay

P1: No, I do eat for comfort, absolutely I do.

This response, an admission of emotional eating, was a rare moment of transparency from Louise. At times, she would reflect on her tendency to disengage from the interview itself or the HLM-ABC program when it felt too intense. Louise’s shifting between these contrary narrative stances, i.e., humour versus seriousness, never quite resolved or funnelled into a clear narration of change. In essence, while Louise openly acknowledged certain gains, these were quickly overridden by her detailing of circumstances that remained unchanged and unchangeable (i.e., her diseases and her survivor identity).

Louise narrated a non-linear story of immutability in relation to lifestyle change, portraying herself as a passive protagonist who was sarcastic, “blunt” and “self-deprecating,” and who often cracked jokes about her “grim” situation. For instance, a self-description of “I am too heavy, too short, fat and ugly” was stated in a jocular way, alongside a perceived invitation for her audience (the interviewer) to laugh at this comment. So too did this brand of humour manifest during group sessions, when she joked with the other women about her favourite food: “cancer-causing hotdogs” and when she jested about whether she could bring a snack of potato chips to the next group session. The interviewer (SY) was admittedly responsive to Louise’s humour, laughing along with her when appropriate. However, an underlying tone of seriousness that came through at important moments was still noted. When for example, certain interview questions provoked more serious responses and Louise was forced to abandon her wry humour, she would express that she had reached her “mental limit.”

Interestingly, by the end of the interview series, Louise declared that “food is not the boss of me” diverging from the “lazy” and passive self she mainly portrayed. The research authors saw subtle narrative transitions and arcs, and in brief spurts she abandoned her humour to reveal willingness to express more vulnerability, and thus hint at her underlying fear and pain. This change in self-awareness was perhaps Louise’s biggest change in characterization in the HLM-ABC; although she did not necessarily see these moments of insight as notable achievements, the researchers did.

#### Felicia

Felicia’s narrative progression, like Louise’s, focused around her identity as being stable rather than on change and progress. Upon starting the group, Felicia mentioned her main purpose for joining the HLM-ABC program was to maintain her health in order to be well for her daughter, and to reduce her risk of cancer recurrence. Her efforts seemed, however, to be thwarted by her persistent view of herself as an overweight individual often referencing her struggles with weight prior to cancer. As such, a narrative tone of hopelessness came through when she reflected on her participation in the program. Felicia’s narrative bore a strong resemblance to Louise’s in terms of the 'narrative foreclosure' implied in Louise’s use of phrases like “my default” (in relation to her lifestyle habits) and “historically I was a bad eater.” Felicia's language was suggestive of someone for whom a lack of satisfaction was part and parcel with her identity. For example, when asked in the mid-way interview if she experienced any new insights about herself and her body vis-à-vis healthy living, her reply illustrated her self-concept as fixed, and the futility of attempting to intervene:

Um [long pause] I don’t know how to put this, I think it’s just when I have done group or you know individual therapy in the past, it’s clear that you are always yourself no matter where you go, um and that, and that there is kind of no magic, no magic bullet to change some of the things you struggle with.

The richness and irony of Felicia’s “magic bullet” metaphor appears to be two-fold. Not only does it capture the current health trend of consuming blended drinks or “smoothies” (epitomized in North America by advertisements for the “Magic Bullet” kitchen appliance and the promises of well-being), and acknowledge that there is no immediate or effortless solution to weight reduction, but the word “bullet” itself harkens to the deep wounds of her cancer diagnosis that seem to have profoundly penetrated her view of self.

The fixedness of weight and the narrative foreclosure expressed in Felicia’s story were, in her telling, connected to her family’s relationship with food. A “master” narrative (Randall et al., 2015) running through her story depicted the act of eating and food preparation as a pleasurable aspect of her family’s culture. She stated that her twin sister is a professional chef and that her mother is an excellent cook: “My mom always jokes that our family runs on its stomach.” Felicia again emphasized the importance of food within the fabric of her family life by stating that “food is a concrete way to take care of people because everyone needs to eat. So if, you know, you can make somebody food, you are able to take care of them in a very basic way.” The nurturing function of food maintained itself throughout Felicia’s narrative, but when it came to increasing her physical activity with the intention of improving her body image, these ideas from the HLM-ABC felt overly burdensome and something that she tended to avoid.

Felicia’s avoidance was explicit throughout her narrative. She called herself a “champion avoider” of her body and weight difficulties. She verbalized her consistent aversion to body image topics in the pre-treatment interview:

I would avoid things that made me deal with my body so I would you know, I didn’t like going shopping for clothes. I don’t … I got better at it as I got older but um [sighs] like yeah if I could ignore my body and concentrate on my brain, then I would.

Felicia kept her responses very short on topics that she would typically avoid, politely yet directly signalling to the interviewer that her avoidance strategies were there to stay, at least throughout the HLM-ABC. The above quote also sheds light on Felicia’s autobiographical reasoning as a cognitively-oriented, intellectual characterization. She seemed to have hoped that the HLM-ABC would provide her with “mental tools” so that she could “trick” herself into establishing healthier lifestyle habits. In contrast, there seemed to be a dearth of emotional language in her narrative, which was told from a rational, intellectual perspective; In essence, Felicia persisted throughout in her inclination to privilege her thoughts over her emotions. Thus, Felicia’s self-identified changes during the HLM-ABC program amounted to mainly restructuring of her thoughts about her body and a newfound sense of keeping some of the HLM-ABC principles (such as engaging in short movement breaks from deskwork) “top of mind.” She also spoke of adopting a more compassionate attitude towards herself, for instance, when she said in the post-treatment interview, “I find it easier to not beat myself up about making choices that are not so good, because it’s not just, it’s not just me [who is struggling].” Felicia’s guilt around her food choices waned when she witnessed the other women struggling to form and sustain new lifestyle habits as well.

Overall, Felicia’s sense of stagnancy and fixed identity as being overweight *and* a breast cancer survivor led to few narrative arcs and a non-linear progression throughout her HLM-ABC journey, which did not amount to a sense of satisfaction with the program. In one aspect, her narrative structure did have a defined beginning and middle (shift in thinking), but did not possess a clear end that would suggest she felt able to make tangible behavioural changes.

### Plot-driven narratives: Melissa and Nicole

#### Melissa

Melissa’s narrative followed a clear change trajectory in the form of a “voyage and return” story. She expressed in her interview series that she felt she was “not anywhere where I was before I had cancer” in reference to her physical health. She expressed what seemed to be a loss of control over her lifestyle choices after finishing her treatments and opted to partake in the HLM-ABC program in order to reclaim her pre-diagnosis self. The way she perceived herself before her breast cancer was as a healthy individual who felt satisfied in her body. She drew comfort from her regularly scheduled exercise classes and workout routine. However, post BC treatment, Melissa felt that she was at “ground zero.” She presented this plotline by stating that she could not return to her past habits and yet she lacked insight into why. Her participation was therefore centred around self-discovery and moving back to a recognizable self—which included being an individual with the ability to efficaciously control her weight.

In addition to struggling with motivation, Melissa commented on how her post-surgery body and the loss of her breast had impacted her self-esteem:

You know, I’m sort of struggling with that too, the idea of body image, because my body’s changed. Um and I probably struggled a bit more with that than the weight thing, because the weight thing I feel like if I just focus and get motivated in everything then I can just lose weight, but where my mastectomy and my reconstruction are concerned you know, I will always have these scars.

Interestingly, Melissa’s perception of her alterable weight as distinct from her overall bodily sense, and fixed physical appearance, seemed to help her in overcoming some of the challenges to her body image. Specifically, the number on the scale representing her weight was not as important to her as the way she *felt* in her body. This self-conceptualization was multi-dimensional and rich, adding a complex quality to her overall narrative. Melissa’s narrative was also characterized by a high level of self-awareness as was apparent in her pre-treatment interview:

I know I can do it, I just somehow lack that motivation or I don’t know what it is and sometimes I question you know is it, you know, ‘Do I not want to do this?’ Like I want to do it, but do I not want to do it? Am I sabotaging myself [laughing] in my mind? I don’t know.

Melissa appeared to engage in active meaning-making during her interview series. She was transparent about her inner dialogue and used metaphors and “thick descriptions” (Randall et al., 2015) to describe her journey. For example, with a sense of nostalgia for her pre-cancer life and body, Melissa invoked the symbol of “old jeans” hanging in her closet a few times, which represented the person she was before her cancer. She described the comfort she used to feel in her jeans, coupled with her motivation to retrieve this lost part of herself:

I have a lot of nice clothes that, you know, I want to wear. And I just saw something on TV and this girl is just wearing like a plain black turtleneck with skinny jeans and I’m like, “Wow, that was what I used to wear and I have that whole outfit upstairs but I can’t fit into those jeans so. You know and that kind of makes you feel sad, but it motivates me. It’s like, that’s what I want to do, I want to be able to just slip on a pair of jeans and t-shirt, because that’s more of how I was.

Quite swiftly, by her mid-way treatment interview, Melissa seemed to feel like she was making headway with her lifestyle goals. She conveyed her enthusiasm for a fitness class she had joined for cancer survivors. At this juncture, there was a noticeable shift in her narrative tone from ambivalence to a more energized and enthusiastic one. By her three-months post-treatment interview, Melissa had transitioned from apathy to activity, clearly pinpointing the reasons for her formerly misunderstood lack of motivation. She drew on her past weight loss stories as a source of present encouragement. She stated:

I just say, ‘Forget it, I’ll never be that size again’ and I refuse to believe that because I know I have two examples - that being [during] university and I started exercising and lost the weight, and after my son, it took a lot longer for me to get there but as soon as I got a trainer I started exercising and lost the weight, so I know it can be done.

Melissa’s consideration of her past self to orient and inform her present self is evidence of “narrative openness.” The above quote also illustrates the sense of personal agency she leveraged to regain what she felt she lost—that is, both her healthful lifestyle and her commitment to that lifestyle. Finally, Melissa revealed her “aha!” moment during her post-treatment interview that inspired her to get back to the self she felt she lost:

I understand now what my problem was and that was really that inner rebel stopping me … And then that’s when you get, you know, the walls kind of go up and be like ‘Whoa!’ like there’s someone telling me to do it. And then it’s like, ‘Well I don’t want to do it,’ cause someone’s saying I *have* to do it, kind of thing … You know, now, because it’s a health thing and everything else, I feel like it’s an obligation to do it, not a choice.

Overall, Melissa’s narrative was linear and plot-driven in how it began with a clear problem, moved to an interim stage of self-reflection and problem-solving, and eventually reached a denouement as evidenced by her adoption of the HLM-ABC principles, and the feeling that she was closer to the self she recognized before cancer.

#### Nicole

Nicole’s narrative was also plot-driven in her telling of her perceived successful change story. Nicole had defined goals from the outset and she appeared to follow a clear path to feeling good in her body and improving her low mood. Nicole considered herself a “normal weight person” even post-treatment (after gaining 5 pounds). Therefore, weight loss was not her main focus. Rather, Nicole sought out the HLM-ABC for social support and the companionship of other BC survivors. Her need for interpersonal contact and to feel accepted was driven by the abandonment she had felt by her friends during and after her treatment. As Nicole described it, her friends referred to her as no longer “normal”, making her feel stigmatized, as a diseased individual in their eyes. She stated that her friends treated her as if she were a completely different person after cancer. In her words, they “deserted me, they don’t call me, they don’t email me. They think I’m a cancer patient.” Despite these pronounced feelings of alienation, Nicole was committed to improving her health habits in order to stave off a cancer recurrence and to feel healthier on a day-to-day basis. Thus, although she joined the HLM-ABC to obtain social support, she also became invested in making health behaviour changes.

For Nicole, feeling healthy was synonymous with feeling “normal.” It seemed there was a part of Nicole that internalized her friends’ perception of her as a “sick” individual. In the mid-way treatment interview, Nicole stated:

I feel like a normal person attending the group. If not, I feel like a sick person. If I stay home for too long, only surrounded by my needy family members, I feel very bad. But then if I draw on some social activities like [the group] and then I feel like a normal person, too.

Importantly, Nicole’s sense of personal and narrative agency became apparent as she narrated how she was coping and overcoming her main issues, which included social isolation, mood difficulties, and “chemo brain” that made her feel forgetful and lethargic. Nicole conveyed that the cancer left a permanent stamp on her and although she was hopeful that her symptoms would dissipate over time, she was currently experiencing an identity change as a cancer survivor, and adjusting to the loss of her breast. She stated:

I feel kind of bad because … I have [had a] mastectomy and I feel like I’m only half a woman now. So my image is not so good. So if I am at home I just dress in cancer clothing. But if I am going out to see other people, then I need to put on special clothing to pretend that I am a normal woman … (*I: That must be very difficult)* … I feel not so bad about it, I just accept it as it is because some people lost their legs, some people lost their arm and some people lost important organs, but then I kind of say to myself ‘that’s no big deal.’ I just lost one side of the breast, I hope I can keep the other side healthy.

Nicole seemed to convey embarrassment about her body, which could have been related to her social withdrawal and feelings of isolation. Her narrative telling was complex and “double-edged” in that her self-portrayal as an isolated person seemed to jointly serve as a barrier to making health behaviour changes (keeping her away from others and disengaged) *and* as a source of motivation to enrol in a structured group with other BC survivors (in order to socialize and work alongside others to achieve similar health goals).

Nicole’s narrative is plot-driven in that she developed clear and tangible insights about herself and these were discussed in relation to her ability to make concrete behavioural changes. For example, Nicole gained awareness about what she termed the “mood-food relationship.” She explained that she is “easily affected by my mood. So if I can manage my stress, my anxiety, and my mood better, then I may actually master better eating habits.” In general, the latter half of Nicole’s interviews were narrated with positivity and hope for the future. Some of the benefits she spoke of included self-awareness, a motivating drive, “regaining balance” after setbacks or other sources of stress, “a positive attitude towards life after cancer,” and making plans to sustain her habits. For example, if her regular walking schedule was to be disrupted by weather, she would create a plan to walk inside in a mall. Consistent with one of the primary aims of the HLM-ABC program, Nicole reported that she was able to maintain her new habits. In her three-month post-treatment interview, Nicole stated, “I am good at sustaining the exercise program and I now formed good habits of exercise, which I enjoy and cannot do without it.” In essence, her new lifestyle changes helped her to narrate a positive story of finding her way, developing greater purpose, and building a newfound sense of security within herself.

## Discussion

Four BC survivors were interviewed longitudinally, at four distinct times, to grasp a comprehensive understanding of their pathways of lifestyle change as they partook in a structured healthy lifestyle intervention. Despite the plentiful weight-loss outcome research in this area, there remains a gap in our knowledge of experiential and psychosocial barriers and facilitators to health behaviour change and weight modification after BC. The current research, by virtue of adopting a narrative analytic lens, elucidates the meaning and process of lifestyle change in the BC survivorship period.

Participant’s storying was characterized as “plot-driven” when their narratives featured change *throughout the storyline* as articulated by the women. Alternately, participant narratives were deemed by the researchers to be “character-driven” when they possessed predominant themes of non-change *within the narrator’s sense of self*, seemingly reflective of fixed character traits or states. That is to say, plot-driven narratives were associated with greater improvements as perceived by the participants, and character-driven narratives were associated with self-reported difficulty or resistance to grasping or applying the core habit-forming tenets of the HLM-ABC program. Being attuned to the participants’ process of storying their experiences revealed that that those women (Melissa & Nicole) who narrated plot-driven experiences seemed to report relative success and described changes in their lifestyles in the direction of healthier habits and greater self-compassion. In contrast, those whose stories were character-driven (Louise and Felicia) appeared to report relatively less success in the intervention and seemingly exhibited less motivation for change. In essence, plot-driven and character-driven narratives seemed to be connected to different change trajectories within the intervention.

Interestingly, we also found that in addition to being associated with poorer self-reported lifestyle change, character-driven narratives also appeared to be storied by women who experienced pre-morbid weight challenges. Louise and Felicia (who stated they struggled to maintain a stable weight before their cancer diagnosis) alluded to how their previous unsuccessful weight loss attempts or stories affected their degree of engagement and/or motivation in the HLM-ABC intervention. It is as though Louise and Felicia’s present narratives represent part of a larger series of similar historical experiences based on recurring plotlines characterized by stability and lack of resolve, contributing to a more resigned, helpless, or hopeless narrator tone. Comparatively, Melissa and Nicole, who did not report pre-morbid weight concerns or weight-loss difficulties, fared better in the program and were more successful in their application of the HLM-ABC principles, which was associated with more notable weight modification experiences (i.e., plot changes). Seeing as previous experiences with weight loss informed these womens’ experiences to a heavier degree than anticipated by the researchers, the importance of assessing for an individual’s history of weight loss or prior behaviour modification successes may be indicated before formal, structured programs.

Our findings relate to research conducted by Green et al. (2014) who demonstrated that cancer identity and self-efficacy for BC and prostate cancer survivors were associated with physical activity and wellness behaviours. They found that if these survivors believed they were capable of affecting change in their cancer outcomes, this sense of self-efficacy translated to improved fitness. Similarly, the incorporation of social cognitive theory into lifestyle initiatives among cancer survivors, which includes a pre-understanding of one’s motivation and barriers and facilitators to health-promotion, has shown success in the direction of improved behaviour change (Stacey et al., 2015). Taken together with our findings, paying attention to the way in which BC survivors articulate their change processes from a narrative perspective provides a window into their self-efficacious beliefs and may provide insight into their level of readiness for health behaviour modification. It thus follows that gauging one’s level of motivation for change and supporting self-efficacy is necessary to bolster success in healthy lifestyle interventions. Therefore, the inclusion of motivational interviewing and psychoeducation on personal health beliefs is an important recommendation for program development (Clifford & Curtis, 2015).

Furthermore, Corbett et al. (2017), in their review of web-based interventions for cancer survivors, have shown that harmonizing the intervention with the unique person is important for adherence and uptake. Tailoring survivors’ individual needs to programs and ensuring that a baseline level of autonomy is present before enrolling in the intervention might be an important avenue for exploration. Additional counselling or individual therapy may be needed prior to enrolling in a group healthy lifestyle program if survivors express or show initial hesitance to establishing new or different lifestyle habits. It seems to be that matching one’s level of readiness and establishing goals consistent with one’s sense of self-efficacy may lead to movement in the direction that one is aiming for, rather than pre-imposed goals that may not necessarily be aligned with or obtainable for some BC survivors.

A limitation of this study includes a lack of transferable study sample. The longitudinal multiple-interview structure among a smaller group of participants allowed for more in depth examination of each narrative case study (Yin, 2014), but cannot be generalized to all BC survivors who participate in healthy lifestyle interventions. As well, the questions that were used in the interview guide were open-ended and non-leading to encourage free and genuine authorship; however, seeing as the HLM-ABC was also being piloted among these women, some of the questions were specific to program evaluation purposes. The authors attempted, to the best of their ability, to extract the narratives and separate out any program evaluation data that was not directly relevant to this multiple-case study. As well, apart from Kohler-Riessman’s method (2008), we employed Randall’s detailed model for narrative analysis (2015), which was originally developed with an ageing population. To our knowledge, this framework for narrative analysis has not been previously used with a cancer population, yet the depth and nuance that it lends to narrative analysis was very fitting for the research aims of this study.

Overall, this study delves deeply into the idiosyncratic experiences of lifestyle modification among a group of BC survivors. With a narrative analysis approach- variation in experience, ranging from poor to successful lifestyle change- was elucidated. The women were able to articulate what was propelling them forward and what was holding them back, despite their shared mutual goals of weight maintenance and improved quality of life. The plot-driven and character-driven narratives represent potential sketches or profiles of individuals that healthcare providers may come across when aiding BC survivors with weight management or lifestyle change. This knowledge has the potential to inform providers about different styles of self-efficacy and personal beliefs surrounding weight loss and may shape the way in which they approach these clinical issues. This multiple-case study may also prompt researchers and clinicians to reflect on the value of moving away from general prescription of weight loss after cancer towards more holistic and individualized health conversations and recommendations in the survivorship period.
